# Deep Sea Water-Dissolved Organic Matter Intake Improves Hyperlipidemia and Inhibits Thrombus Formation and Vascular Inflammation in High-Fat Diet Hamsters

**DOI:** 10.3390/life12010082

**Published:** 2022-01-07

**Authors:** Chia-Chun Wu, Yu-Hsuan Cheng, Kuo-Hsin Chen, Chiang-Ting Chien

**Affiliations:** 1 Department of Life Science, School of Life Science, College of Science, National Taiwan Normal University, Taipei 11677, Taiwan; 60642026s@ntnu.edu.tw (C.-C.W.); 80943003s@ntnu.edu.tw (Y.-H.C.); 2 Department of Surgery, Division of General Surgery, Far-Eastern Memorial Hospital, New Taipei City 22056, Taiwan; 3 Department of Electrical Engineering, Yuan Ze University, Taoyuan City 32003, Taiwan

**Keywords:** hyperlipidemia, deep sea water-dissolved organic matter, non-alcoholic fatty liver disease, atherosclerosis, apoptosis, platelet adhesion, von Willebrand factor, intercellular adhesion molecule-1, inflammation

## Abstract

Non-alcoholic fatty liver disease (NAFLD) is a chronic liver disease caused by oxidative stress, inflammation and lipid deposition within liver cells, and is subsequently contributing to cardiovascular diseases such as atherosclerosis. Deep sea water (DSW) is characterized by its clearance and abundant nutrients with antioxidant and anti-inflammatory activity to confer therapeutic potential. We aimed to explore the therapeutic capability of our prepared multi-filtration DSW-dissolved organic matter (DSW-DOM) on high-fat diet-induced hyperlipidemia and endothelial dysfunction in hamsters. A high-fat/high-cholesterol diet led to increased oxidative stress, including blood reactive oxygen species (ROS), plasma malondialdehyde (MDA) and hepatic CYP2E1 expression; an increased hyperlipidemic profile and SREBP 1-mediated fatty liver; promoted NFκB p65-mediated hepatic inflammation; triggered PARP-mediated hepatic apoptosis; and enhanced endothelial intercellular adhesion molecule-1 (ICAM-1) and von Willebrand factor (VWF)-mediated atherosclerosis associated with the depressed hepatic antioxidant Paraoxonase 1 (PON1) expression. The DSW-DOM-enriched 1295 fraction, with strong H_2_O_2_ scavenging activity, efficiently reduced several oxidative stress parameters, the lipid profile, inflammation, and apoptosis, possibly through the PON1-mediated antioxidant capability. Furthermore, DSW-DOM treatment significantly decreased the endothelial ICAM-1 and VWF expression, subsequently leading to the elongation of time to occlusion of FeCl_3_-induced arterial thrombosis and to the inhibition of FeCl_3_-induced fluorescent platelet adhesion to mesentery arterioles in the high-fat diet. Based on the above results, our data suggest that DSW-DOM intake via antioxidant defense mechanisms confers protective effects against high-fat diet-enhanced, oxidative stress-mediated hyperlipidemia, and endothelial dysfunction evoked atherosclerosis by downregulating oxidative injury, lipogenesis, inflammation and apoptosis.

## 1. Introduction

Hyperlipidemia is a well-known risk factor for cardiovascular diseases (CVDs) and can be classified as either familial or acquired. Acquired hyperlipidemia comes from unbalanced eating habit or metabolic disorders that lead to alterations in plasma lipid and lipoprotein metabolism [1]. High blood cholesterol and triglyceride (TG) levels are commonly considered as leading biomarkers of hyperlipidemic processes [2]. Increased blood cholesterol, such as low-density lipoprotein cholesterol (LDL-C), very low-density lipoprotein cholesterol (VLDL-C) and total cholesterol (TC), leads to a high risk of hypertension, atherosclerosis and other CVDs [3,4,5]. Further, blood cholesterol levels are highly associated with hepatic lipid metabolism since cholesterol is synthesized in the liver. Accordingly, hepatic lipid metabolism is an important target to evaluate the pharmacological effect on hyperlipidemia [6].

Non-alcoholic fatty liver disease (NAFLD) is one type of fatty liver that is not associated with excess alcohol consumption [7]. The exact cause of NAFLD is not clear, yet it appears to be related to insulin resistance, mitochondrial dysfunction, inflammation and oxidative stress [8]. NAFLD is a hepatic manifestation of metabolic syndrome (MS) characterized by considerable triglyceride accumulation [9]. Diabetes, elevated cholesterol and TG levels are the risk factors of NAFLD [10]. NAFLD may develop a series of diseases, which ranges from steatosis to nonalcoholic steatohepatitis (NASH), cirrhosis and even hepatocellular carcinoma (HCC) [11]. The global prevalence of NAFLD is approximately 25.24% at present [12], hence there is a serious need to solve problems resulting from NAFLD. However, there is no effective drug available for NAFLD right now [9].

Atherosclerosis is a lipid-driven chronic inflammation with leukocytes recruitment and endothelial dysfunction in large and medium-sized arteries [13]. Endothelial dysfunction is one of the earliest events in atherosclerosis, including disruption of antiplatelet mechanisms [14]. Smoking, elevated low-density lipoprotein (LDL), reduced high-density cholesterol (HDL) and insulin resistance trigger platelet activation [15]. Platelet-released proinflammatory factors promote monocyte recruitment, adhesion molecules expression and ox-LDL uptake [16]. Ox-LDL acts as a stimulator of the immune response, which recruits monocytes migration and promotes macrophage transformation. Macrophages engulf ox-LDL and turn into foam cells, which further trigger atherosclerotic lesions [17]. According to statistics, atherosclerosis is responsible for 50% of deaths in the modern society [18].

Deep sea water (DSW pumped from a depth over 200 m is characterized by cold temperature, abundant nutrients and is pathogen free. Due to its high productivity and large quantity, the potential of DSW in agriculture, cosmetics and health improvement gained more attention worldwide [19]. DSW was reported to have therapeutic effects on atherosclerosis [20], atopic eczema/dermatitis [21], diabetes and obesity [22], hyperlipidemia [23], hypertension [24], liver steatosis [25] and hyperglycemia [26]. DSW has demonstrated its efficacy on lowering the TC, LDL and lipid peroxidation in hypercholesterolemic humans [27] and rabbits [20,23]. DSW intake resulted in higher fecal cholesterol and bile acid excretions, and hence decreased TC levels [28]. DSW is also capable of improving the lipid profiles in the serum and liver of animal models [23,29,30]. Previous studies evidenced that DSW decreased the hepatic lipid concentration through activating AMP-activated protein kinase (AMPK), thus inhibiting cholesterol and fatty acid synthesis [31]. The effect of DSW in ameliorating the lipid profile suggested the potential of being a hypotensive and lipid-lowering agent for preventing or treating CVD, such as atherosclerosis [32]. Atherogenesis implies the formation of plaque, which is composed of fat, cholesterol, cellular waste products, calcium and other substances. Prevention of atherogenesis can avoid coronary artery disease and stroke. In earlier studies, DSW with a hardness of 300, 900 and 1500 significantly decreased the atherogenic index (TC—high-density lipoprotein cholesterol (HDL-C)/HDL-C) [28] and prevented the atherogenesis process [20,30]. DSW inhibits cardiovascular diseases by decreasing the TC, TG, atherogenic index, and MDA levels while increasing the serum Trolox equivalent antioxidant capacity (TEAC) [32] and upregulating hepatic LDL receptors (LDL-R) [28]. The cardioprotective effects of DSW was further proven as it reduces abnormal cardiac structures, apoptosis, and cardiac survival signaling, and enhance insulin-like growth factor 1 receptor (IGF-1R) [33]. DSW also improved cardiovascular hemodynamics via its minerals [24]. Based on the above information, these studies suggest that DSW has several potential therapeutic applications, primarily through the action of Mg^2+^.

Deep sea water-dissolved organic matter (DSW-DOM) is the organic matter that passes through a sub-micron pore-sized filter. Previous studies showed that DSW-DOM from the Cape of Muroto (Latitude 33.5° N and Longitude134.10° E) inhibited platelet aggregation and activity of cyclooxygenase 1 significantly [34]. Moreover, DSW-DOM increased the expression of heme oxygenase-1, an anti-atherogenic molecule, in endothelial cells. Since DSW-DOM feeding delayed the progression of atherosclerosis remarkably, it was considered a future therapeutic strategy [34]. Previous studies showed that the lipoprotein profile and lipid metabolism of hamsters are similar to humans [35], including a large proportion of the non-HDL form circulating lipoproteins, possessing cholesteryl ester transport protein, receptor-mediated uptake of LDL [36]. In addition, hamsters with a high-cholesterol or fat-rich diet can develop obesity, insulin resistance, hypercholesterolemia and atherosclerosis in a short period of time [36,37,38]. As mentioned above, these features indicate that the hamster is a proper animal model for the assessment of drugs on weight loss, glucose tolerance, hypertriglyceridemia and hypercholesterolemia [39]. In the present study, we aimed to determine the potentially therapeutic effect and mechanism of our prepared novel DSW-DOM on the profile of hyperlipidemia, oxidative stress, inflammation and atherosclerosis in high-fat diet hamsters.

## 2. Materials and Methods

### 2.1. Animals

A total of seventy-four Golden Syrian hamsters (male, 6 weeks old) were purchased from the National Laboratory Animal Center (Taipei, Taiwan) and fed with a chow diet for 7 days for acclimation. All animals were housed in the Experimental Animal Center of National Taiwan Normal University, in a room kept at 22–27 °C, 50–60% relative humidity, a 12-h light/dark cycle and free access to food and water. The food and water intake and feces output were monitored by a metabolic cage. All surgical and experimental procedures were all approved by the Institutional Animal Care and Use Committee of National Taiwan Normal University, with Approval Number 105018 (on the date of 2016 September 6), and were in accordance with the guidelines of the National Science Council of the Republic of China (1997).

### 2.2. Grouping

After acclimation, animals were randomly assigned into four groups: the control group (CON, *n* = 16, 8 for acute arterial thrombosis and 8 for platelet adhesive assay) were fed with a normal diet (D12102C, 10% kcal fat; Research Diet, New Brunswick, NJ, USA); high-fat/high-cholesterol diet group (HCD, *n* = 16, 8 for acute arterial thrombosis and 8 for platelet adhesive assay) were fed with a high-fat/high-cholesterol diet (D12108C, 40% kcal fat with 1.25% cholesterol; Research Diet); and groups in the high-fat/high-cholesterol diet with a low or high dose of dissolved organic matter (DOM) from a C18 membrane (HCD-LD, *n* = 16, 8 for acute arterial thrombosis and 8 for platelet adhesive assay; HCD-HD, *n* = 16, 8 for acute arterial thrombosis and 8 for platelet adhesive assay) for 8 weeks.

### 2.3. Preparation of Deep Sea Water-Dissolved Organic Matter (DSW-DOM)

**Figure 1:**
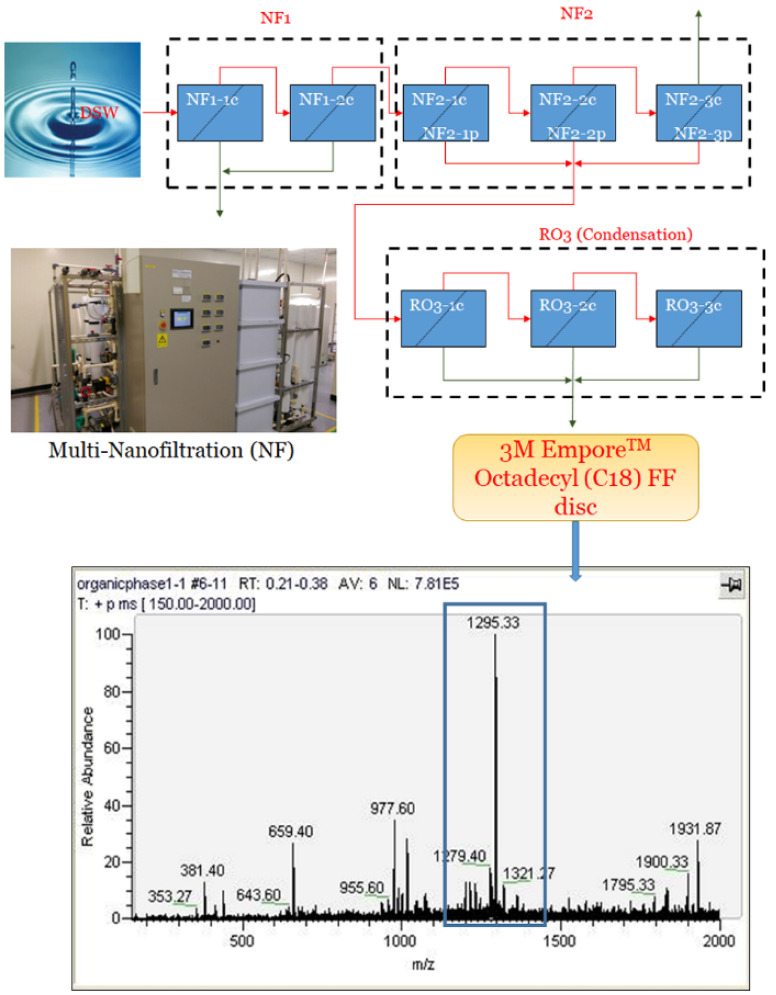
The preparation and analysis of DSW-DOM. The original DSW was passed through a multi-nanofiltration system to obtain the condensed DSW, and subsequently passed through a C18 membrane to extract the DSW-DOM from the C18 membrane. The analyzed DSW-DOM contained the major component 1295 fraction.

The preparation of DSW-DOM was indicated in Figure 1. Original DSW was obtained from a depth of approximately 618 m in Chisingtan Bay, Hua-Lien County, Taiwan. The condensed and desalinated DSW was performed with multi-nanofiltration and provided by Stone and Resources Industry Research and Development Center (Hualien, Taiwan). DSW-DOM preparation followed approximately 2000 L of DSW was passed through a 3M Empore^TM^ Octadecyl (C18) FF disc (90 mm) to keep the organic substance similar to what has been described previously [34]. Then, the C18 FF disc was dissolved in 25 mL of ethanol to acquire filtered-DOM, which was described as DSW-DOM. The dosages of our prepared DSW-DOM to the animals at the values of 100 or 200 μL of DSW-DOM twice a week were according to similar reports by Radhakrishnan et al. [34]. The HCD-HD hamsters were fed with 200 μL of DSW-DOM twice a week. The HCD-LD hamsters were orally fed with 100 μL of DSW-DOM and 100 μL of ddH_2_O twice a week. CON and HCD hamsters were orally fed 200 μL of 50% ethanol and 50% ddH_2_O twice a week. We used a Thermo Finnigan LCQ Classic LC/MS/MS ion trap system to characterize the possible components in DSW-DOM. Our prepared DSW-DOM contained MS 1295 rich fraction as shown in Figure 1.

### 2.4. Hydrogen Peroxide (H_2_O_2_) Scavenging Capacity of DSW-DOM

In this part of study, the H_2_O_2_ scavenging level of the different concentration of DSW-DOM was measured by the luminol-amplification ultrasensitive chemiluminescence detection method as described previously [40]. In brief, 0, 100 or 200 µL of freshly prepared DSW-DOM solution in ethanol was mixed with 0.5 mL of 0.1 mmol/L luminol (5-amino-2,3-dihydro-1,4-phthalazinedione, Sigma, Chemical Co., St. Louis, MO, USA) and 0.1 mL of H_2_O_2_ (0.03%) and was analyzed with a chemiluminescence analyzing system (CLA-ID3, Tohoku Electronic Inc. Co., Sendai, Japan). The chemiluminescence signals emitted from the mixture of DSW-DOM solution and luminol, which represented the hydrogen peroxide content in the mixture, were recorded. The enhanced chemiluminescent signals from the sample–luminol–H_2_O_2_ mixture were recorded for 300 s. The total chemiluminescent (CL) counts were calculated by the area under the curve and were displayed as counts/10 s.

### 2.5. Blood Lipid Analysis

After 8 weeks’ diet, plasma samples were collected in these four groups of animals (*n* = 8) for measuring the TC, TG, LDL and HDL levels.

### 2.6. Triglyceride (TG) Colorimetric Assay

Hepatic and fecal triglyceride was measured in order to explore whether DSW-DOM changes the metabolism of triglyceride in hamsters. Triglyceride was detected by the Triglyceride Colorimetric Assay Kit (No. 10010303; Cayman, Ann Arbor, MI, USA). Minced tissue was homogenized with diluted NP40 Substitute Assay Reagent and centrifuged at 10,000 relative centrifugal field (rcf) for 10 min. The supernatant was then transferred to another tube. The tissue sample was mixed with diluted Enzyme Mixture and incubated for 15 min before detecting the absorbance at 530 nm.

### 2.7. Ferric Chloride (FeCl_3_)-Induced Acute Arterial Thrombosis

FeCl_3_-induced vascular injury is a widely used model in thrombosis research because it allows variable levels of injury in different vascular beds and can be easily monitored. As a consequence, the animal model of FeCl_3_-induced arterial thrombosis has been used extensively to assess the antithrombotic activities of test agents [41]. FeCl_3_ causes oxidative stress, lipid peroxidation and endothelial destruction, resulting in an occlusive thrombus [42]. In order to monitor blood flow and obtain larger tissue sampling more easily, the carotid artery was chosen in the four groups of rats (each group with 8 animals) in this study. A flow probe (Probe# 0.5VB652; Transonic Systems, Inc., Ithaca, NY) cradle in the left carotid artery was used for blood-flow measurements. After 5 minutes’ stabilizing, a 30% FeCl_3_ solution was dropped on the left carotid artery. The adventitial FeCl_3_ solution easily diffused into the arterial tissue, which may harm the endothelium, media, and intima of the carotid artery. This effect, called the Fenton reaction-mediated injury (impairment of nitric oxide release), causes reduced arterial blood flow and even occlusion. The time the arterial blood flow dropped below 0.1 mL/min was defined as the time to occlusion (TTO).

### 2.8. Platelet Adhesiveness Detection

Whole blood was collected from the inferior vena cava of 10 control animals and collected in 1.5-mL polypropylene tubes with 300 μL of heparin (30 U/mL). Platelet-rich plasma was obtained by centrifugation at 1200 revolutions per minute (rpm), 4 °C for 5 min. After centrifugation, we transferred the plasma and buffy coat to fresh microcentrifuge tubes and recentrifuged at 1200 rpm. The platelet-rich plasma was transferred to fresh tubes containing 2 μL of prostacyclin (PGI_2_, 2 μg/mL) and incubated at 37 °C for 5 min. The pellet was resuspended and washed in Tyrode-HEPES buffer (137 mM sodium chloride (NaCl), 0.3 mM sodium hydrogen phosphate (Na_2_HPO_4_), 2 mM potassium chloride (KCl), 12 mM sodium hydrogen carbonate (NaHCO_3_), 5 mM 4-(2-hydroxyethyl)-1-piperazineethanesulfonic acid (HEPES), 5 mM glucose, 0.35% bovine serum albumin (BSA) and 2 μL prostacyclin (PGI_2_)). After a centrifugation at 2800 rpm, the pellet was incubated at 37 °C for another 5 min. In order to remove PGI_2_, the washing steps would be conducted twice. Platelets were labeled with calcein green, acetoxymethyl (AM, 2.5 μg/mL) (C34852; Life technologies, Waltham, MA, USA) for 10 min at room temperature.

The fluorescently labeled platelets (FP) were infused into four groups (*n* = 8) of hamsters (1.25 × 10^8^ platelets/kg) through the jugular vein with PE-10. A total of 300 s later, 20 μL FeCl_3_ was applied onto the mesenteric arteriole (about 200~300 μm diameter) to induce acute artery thrombosis and platelet aggregation. The whole process was monitored in real time by a fluorescent dissecting microscope (Leica DMLFSA, Wetzar, Hessen, Germany) and recorded by The MetaVue System (Molecular Devices, LLC., San Jose, CA, USA).

### 2.9. Blood Reactive Oxygen Species (ROS) Detection

Blood ROS was measured via a Chemiluminescence Analyzer. Luminol was chosen as the CL agent in this experiment to detect ROS.

### 2.10. Malondialdehyde (MDA) Assay

To explore whether DSW-DOM ameliorates hyperlipidemia, the plasma MDA level, a lipid peroxidation product of oxidative stress, was measured. Plasma MDA was detected by an MDA Assay Kit (ab118970; Abcam, Cambridge, UK). The plasma was mixed with 42 mM of sulfuric acid (H_2_SO_4_) and phosphotungstic acid solution. The plasma mixture was incubated for 5 min at room temperature and centrifuged at 13,000 rcf for 3 min. The pellet was collected and resuspended with ddH_2_O and butylated hydroxytoluene (BHT), which were followed by addition of thiobarbituric acid (TBA) solution and incubation for 60 min at 95 °C. The mixture was cooled down to room temperature on ice, before detecting the absorbance at 532 nm.

### 2.11. Histological Analysis

High-fat/high-cholesterol diet-induced pathological change and the effect of DSW-DOM on livers were checked by hematoxylin and eosin staining. Liver was fixed in 10% formalin and delivered to the National Taiwan University Hospital Department of Pathology (Taipei, Taiwan) for paraffin embedding. Sections were cut on a microtome and were deparaffinized in xylene and rehydrated in ethanol. The slides were immersed in xylene for 10 min at room temperature, and then were sequentially bathed in a series of ethanol (each for 5 min) at room temperature.

The slides were stained in hematoxylin for 1 min and rinsed in 0.2% aqua ammonia. Then the sections were counterstained in eosin for 30–60 s and washed in ddH_2_O. The slides were dehydrated in ethanol and mounted in mounting medium (Leica, Wetzlar, Germany).

### 2.12. Oil Red O Stain

Sections were fixed with 10% formalin for 10 min and briefly washed with ddH_2_O. After rinsing in 60% isopropanol, the slides were stained with 0.5% Oil Red O working solution in propyleneglycol for 15 min. Next, the slides were rinsed in 60% isopropanol and ddH_2_O, followed by counter staining with hematoxylin (HHS16; Sigma Aldrich, St. Louis, MO, USA) for 1 min and mounted with a mounting medium.

### 2.13. Immunohistochemistry

The tissue sections were deparaffinized, rehydrated and submitted to antigen retrieval buffer (10 mM Sodium citrate, 0.05% Tween 20, pH 6.0) at 95 °C for heat-induced epitope retrieval. After 20 min, 3% H_2_O_2_ was applied for 15 min to clean endogenous peroxidase. The slides were blocked for non-specific binding with 5% BSA (A7906; Sigma-Aldrich, St. Louis, MO, USA) for 1 h at room temperature and incubated with the primary antibodies for 18 h at 4 °C. Primary antibodies, including rabbit anti intercellular adhesion molecule 1 (ICAM-1, 1:100; bs-0.08R; Bioss, Woburn, MA, USA), rabbit anti poly (ADP-ribose) polymerase (PARP, 1:500; ac19127; Abcam, Cambridge, UK), mouse anti paraoxonase 1 (PON1, 1:500; ab24261; Abcam, Cambridge, UK), mouse anti sterol regulatory element-binding transcription factor 1 (SREBP1, 1:300; sc-13551; Santa Cruz, Dallas, TX, USA) and rabbit anti Von Willebrand factor (VWF, 1:100; 11778-1-AP; Proteintech, Chicago, IL, USA) were used.

Tissue sections were incubated with secondary antibodies horseradish peroxidase (HRP)-conjugated rabbit anti-mouse immunoglobulin G (IgG) or goat anti-rabbit IgG (1:800; Sigma Aldrich St. Louis, MO, USA) for 1 h and washed in phosphate buffer saline Tween-20 (PBST). The slides were immersed in 3′,3′-diaminobenzendine (DAB) (ab64238; Abcam, Cambridge, UK) for 5–10 min and rinsed in ddH_2_O. Then the slides were stained with hematoxylin for 1 min and washed with 0.2% aqua ammonia. The slides were dehydrated in a series of ethanol and mounted with mounting medium (Leica, Wetzlar, Germany).

## 3. Western Blot

Frozen tissues were grinded into powders in liquid nitrogen and dissolved in a radio immunoprecipitation assay (RIPA) buffer (Bio Basic Inc., Markham, ON, Canada) with protease inhibitor (78430; Thermo Fisher Scientific, Rockford, IL, USA) for cell lysis and protein extraction. The tissue homogenate was centrifuged for 30 min at 14,000 rpm and the supernatant was collected. The protein concentration was determined by a bicinchoninic acid (BCA) assay (23228; Thermo Fisher Scientific, Rockford, IL, USA). Protein extract was mixed with 1X sample buffer and boiled for 5 min at 99 °C. Samples were separated on 10% sodium dodecyl sulfate polyacrylamide gel electrophoresis (SDS-PAGE) and transferred to a polyvinylidene difluoride (PVDF) membrane (Millipore, Billerica, MA, USA) over 18 h. After transferring, the membrane was incubated with 5% BSA (ALB001; Bioshop Canada Inc, Burlington, ON, Canada) for 1 h, and incubated with primary antibodies overnight at 4 °C. Primary antibodies including mouse anti cytochrome p450 CYP2E1 (1:1000, AB 1252, Chemicon International, Temecular, CA, USA), NFκB p65 (1:1000, MAB 50781, R&D Systems, MP, USA), rabbit anti PARP (1:500; ac19127; Abcam, Cambridge, UK), mouse anti PON1 (1:1000; ab24261; Abcam, Cambridge, UK), mouse anti SREBP1 (1:1000; sc-13551; Santa Cruz, Dallas, TX, USA) and mouse anti β-Actin (1:5000; A5441; Sigma Aldrich St. Louis, MO, USA) were used. The membrane was incubated with HRP-conjugated secondary antibodies for one hour at room temperature. Signal detection was performed by the enhanced chemiluminescence (ECL) kit (NEL121001EA; PerkinElmer, Waltham, MA, USA).

### Statistical Analysis

Data are presented as the mean ± standard error the mean (SEM), and *p* < 0.05 was considered statistically significant. Statistical evaluation was done by one-way analysis of variance (ANOVA) and Tukey’s multiple comparison test.

## 4. Results

### 4.1. Body Weight was Not Significantly Different among Four Groups of Animals

**Figure 2:**
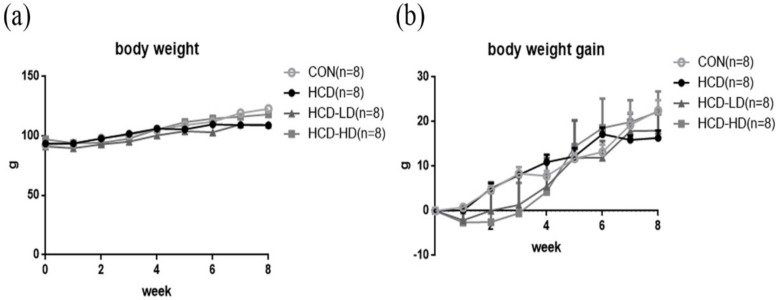
Effect of DSW-DOM on body weight (**a**) and body weight gain (**b**) in four groups of hamsters. Data are expressed as the mean ± SEM (*n* = 8).

Body weight of the hamsters was recorded once a week. At the end of the experiment, there was no significant difference in body weight (Figure 2a) and body weight gain (Figure 2b) between groups. Therefore, a high-cholesterol diet did not affect body weight.

### 4.2. Food Intake and Feces

**Figure 3:**
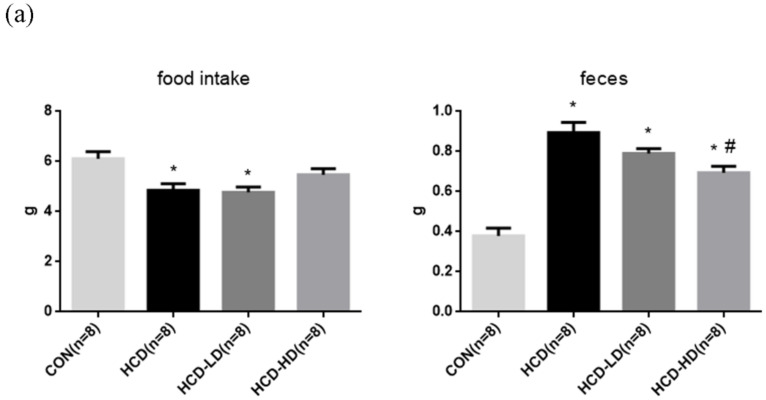

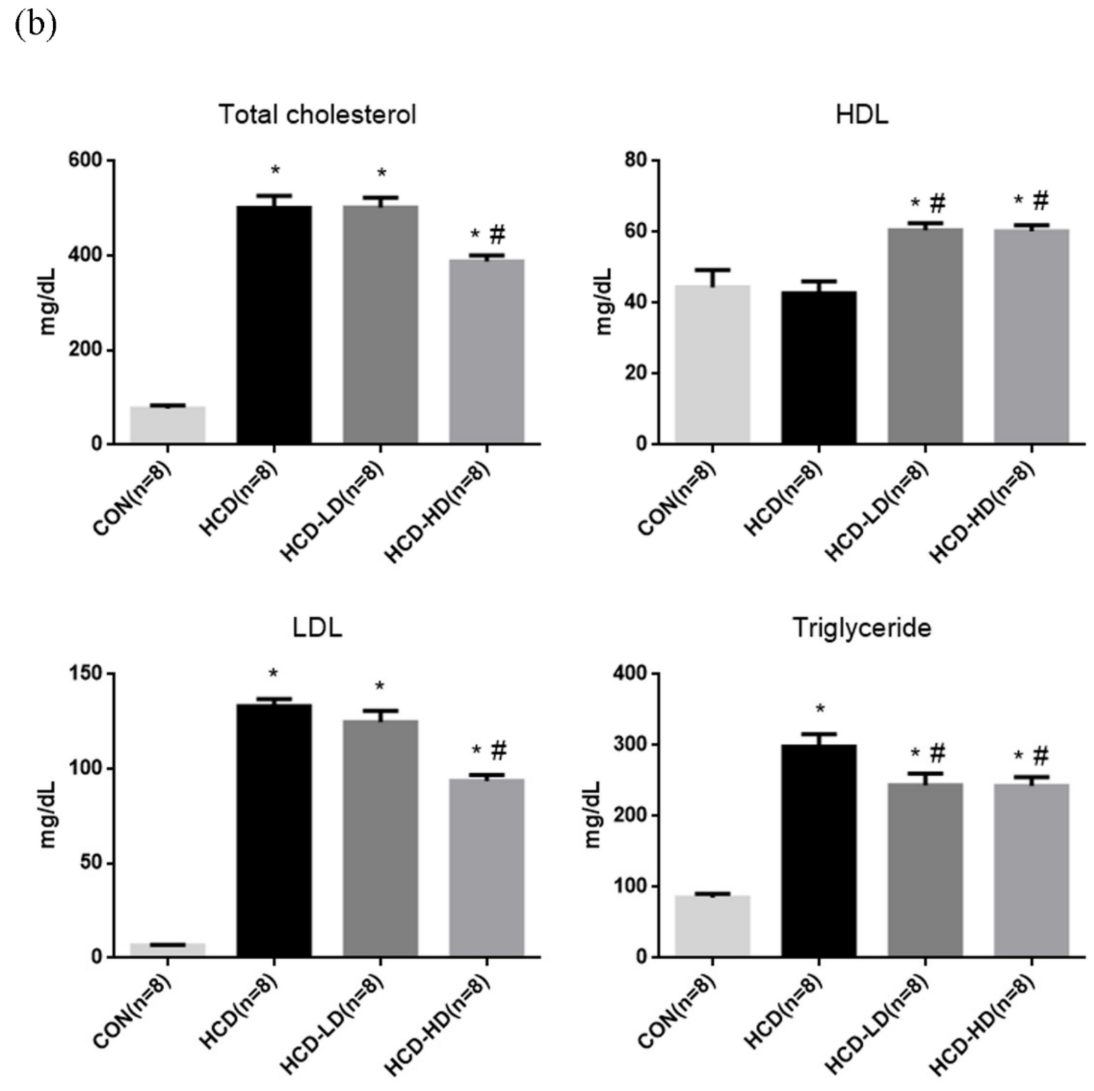
Effect of DSW-DOM on the daily food consumption and fecal weight (**a**) and lipid profile (**b**) in four groups of hamsters. Data are expressed as the mean ± SEM (*n* = 8). * *p* < 0.05 vs. CON; # *p* < 0.05 vs. HCD.

To evaluate the effect of a high-fat/high-cholesterol diet and DSW-DOM on the baseline levels of the physiological parameters, we determined the body weight, body weight gain, food intake and feces amount among these four groups, as shown in Figure 3. The 24-h food intake and feces weight were recorded at the 8th week. Our data indicated that the amount of food intake of the HCD group and HCD-LD group were less than the CON group (Figure 3a). The fecal weight of HCD was considerably increased when compared to the CON group. On the other hand, the HCD-HD group had a much lower feces amount than the HCD group (Figure 3a).

### 4.3. Lipid Profile

After 8 weeks of a high-fat/high-cholesterol diet, plasma samples were collected for lipid profile analysis. HCD showed a significantly increased TC, TG and LDL level. Both a high and low dose of DSW-DOW had significantly reduced the TG level and raised the HDL level. Additionally, a high dose of DSW-DOM had a markedly decreased TC and LDL content (Figure 3b).

### 4.4. Hepatic and Fecal Triglyceride Concentration

Because the weekly record showed that taking DSW-DOM successfully attenuated an increase of triglycerides in the blood, the effects of DSW-DOM on the hepatic lipid concentrations were evaluated. A high-fat/high-cholesterol diet led to accumulating considerable triglycerides in the liver.

### 4.5. DSW-DOM Can Efficiently Scavenge Hydrogen Peroxide (H_2_O_2_) In Vitro

**Figure 4:**
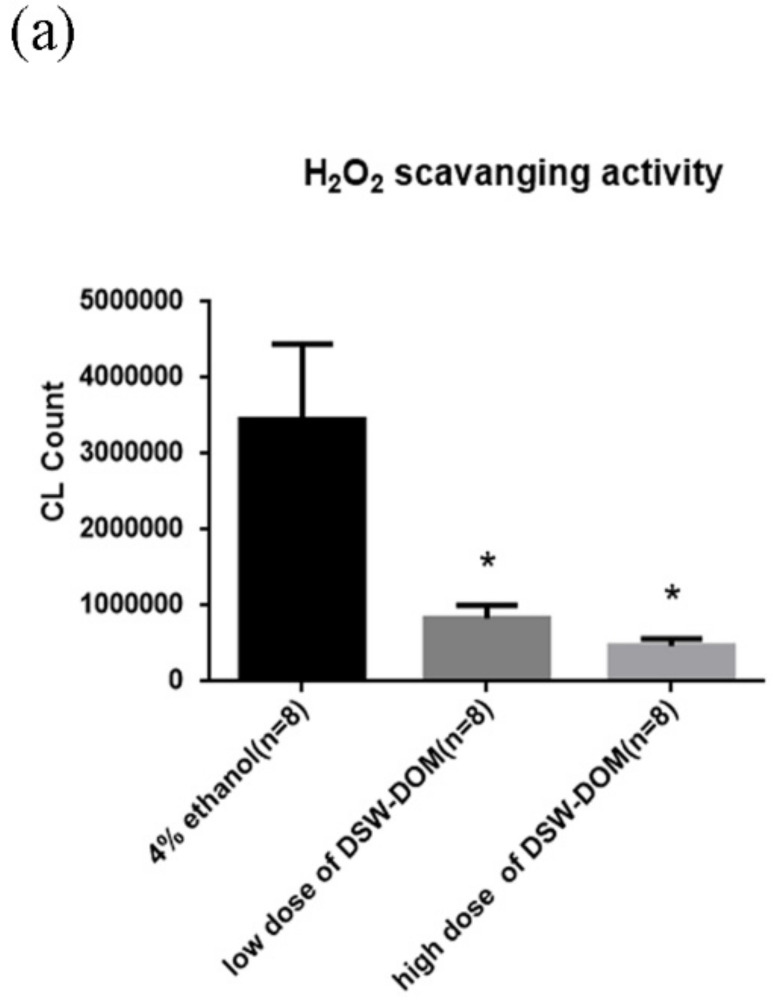

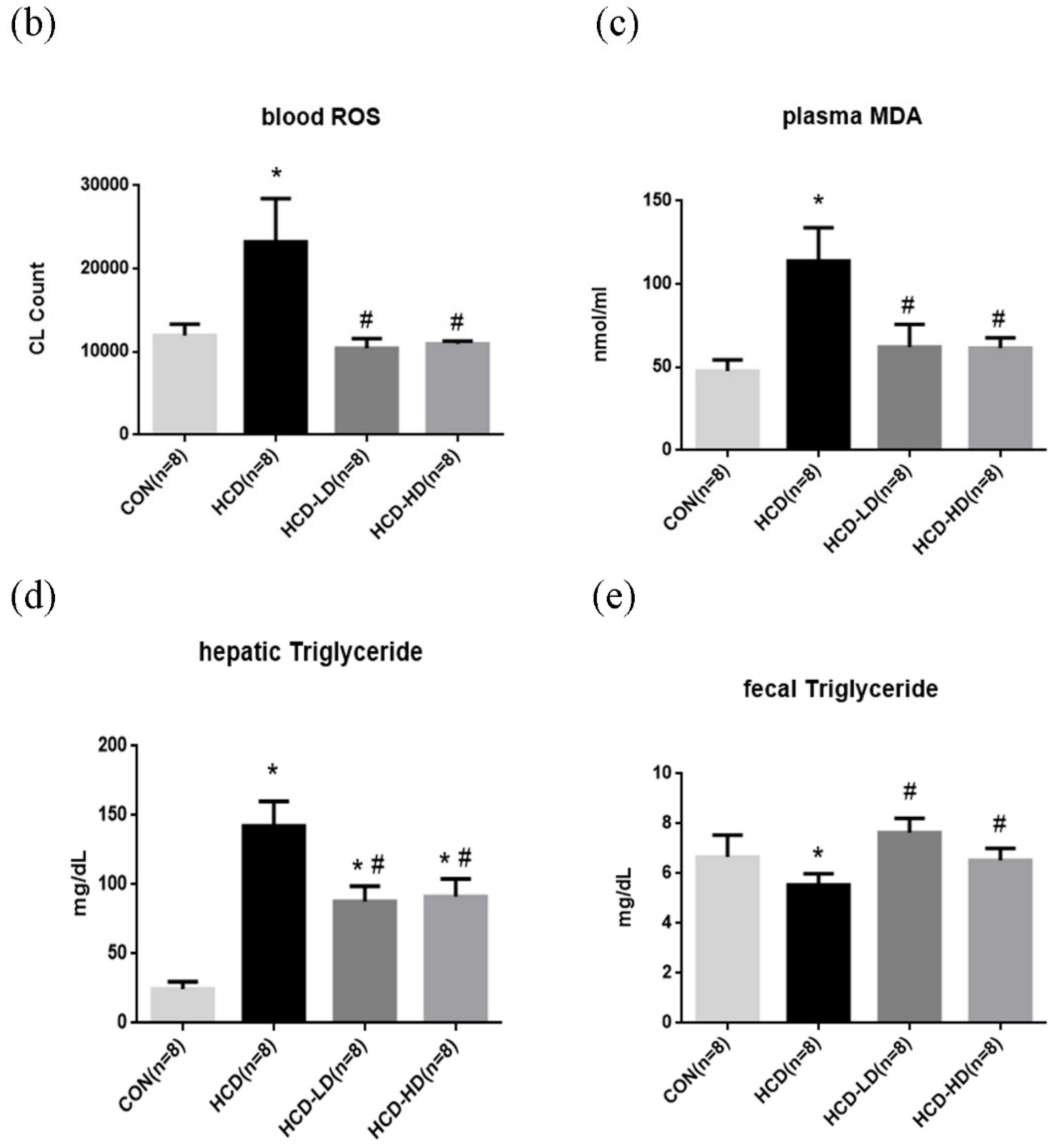
Effect of DSW-DOM on H_2_O_2_ scavenging activity (**a**), blood ROS (**b**), plasma MDA (**c**), hepatic triglyceride (**d**) and fecal triglycerides (**e**) in four groups of hamsters. Data are expressed as the mean ± SEM (*n* = 8). * *p* < 0.05 vs. CON; # *p* < 0.05 vs. HCD.

H_2_O_2_ scavenging capacity of DSW-DOM was checked by a Chemiluminescence Analyzer. Antioxidants are capable of stabilizing or deactivating free radicals. By reducing the energy of free radicals or passing electrons to them, antioxidants make free radicals become stable. Accordingly, antioxidant would present lower CL after adding H_2_O_2_. In the result, both a high and low dose of DSW-DOM showed extremely lower CL counts than the ethanol control (Figure 4a). This result suggested that DSW-DOM was a fine antioxidant.

### 4.6. DSW-DOM Significantly Reduces Blood Reactive Oxygen Species (ROS)

Blood ROS was checked by an ultrasensitive Chemiluminescence Analyzer. CL represented the oxidation level of the sample. In the result, blood ROS was extremely higher in HCD. In contrast, HCD-LD and HCD-HD had fewer ROS than HCD (Figure 4b). HCD greatly raised blood ROS, whereas DSW-DOM consumption markedly eliminated ROS induced by the high-fat/high-cholesterol diet.

### 4.7. Malondialdehyde (MDA) Concentration in Plasma

MDA is the product of lipid peroxidation. In this study, a high-fat/high-cholesterol diet significantly elevated the blood ROS levels. The increased oxidative stress would cause cell damage through lipid peroxidation. As a consequence, quantification of lipid peroxidation is essential to evaluate oxidative damage. Similar to the result of the blood ROS, a high-fat/high-cholesterol diet greatly increased the plasma MDA concentration. On the other hand, the DSW-DOM-treated groups (HCD-LD, HCD-HD) showed a lower level of MDA as compared to HCD (Figure 4c).

### 4.8. DSW-DOM Significantly Reduces Hepatic Triglycerides via the Increased Fecal Excretion of Triglycerides

The hepatic triglycerides in the HCD hamsters was higher than in CON (Figure 4d). Both HCD-LD and HCD-HD presented less hepatic triglycerides (Figure 4d) vs. HCD. To determine whether the reduced hepatic triglycerides originated from the increasing triglyceride excretion, the fecal triglyceride concentration was analyzed. As shown in Figure 4e, the level of fecal triglycerides of HCD-LD and HCD-HD were higher than in HCD. These results revealed that DSW-DOW increased triglyceride excretion, hence leading to decreased lipid accumulation in the liver.

### 4.9. DSW-DOM Significantly Reduces the Oxidative and Inflammatory Parameters

**Figure 5:**
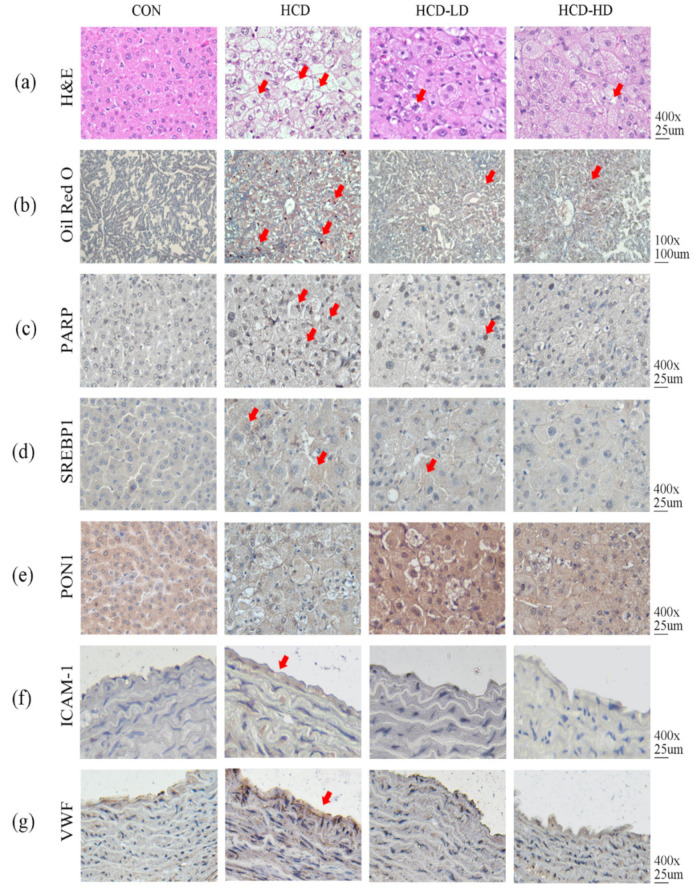
Effect of DSW-DOM on (**a**) H&E stain 400×, (**b**) Oil Red O stain 100×, (**c**) PARP 400×, (**d**) SREBP1 400×, (**e**) PON1 400×, (**f**) ICAM-1 400× and (**g**) VWF 400× in four groups of hamsters. In-creased fat accumulation with red arrows in the fatty liver of HCD hamsters by H&E (**a**) and Oil Red O. (**b**). By contrast, treatment with DSW-DOM treatment showed less lipid droplets in the HCD-LD and HCD-HD livers. The apoptosis marker, PARP (**c**) and lipogenesis marker, SREBP 1 (**d**) expression were significantly increased in HCD vs. CON and reduced expression in HCD-LD and HCD-HD. The PON1, an antioxidant enzyme of HDL expression was decreased in HCD, whereas as the PON1 expression in HCD-LD and HCD-HD showed higher PON1 expression than HCD (**e**). High levels of pro-inflammatory ICAM-1 expressed in the aorta of HCD, yet ICAM-1 was barely seen in HCD-LD and HCD-HD (**f**). For platelet recruitment and adhesion, VWF expression was enhanced in endothelium of aorta, while DSW-DOM decreased VWF expression (**g**). A marked expression and location of the pathologic alteration is indicated with the red arrows.

Under an 8-week high-fat/high-cholesterol diet, the livers of these hamsters turned into fatty livers. Liver sections were stained with hematoxylin and eosin and Oil Red O to assess the fat accumulation. Considerable fat could be observed in the fatty liver of HCD hamsters by hematoxylin and eosin (Figure 5a) and Oil Red O (Figure 5b). By contrast, treatment with the DSW-DOM treatment showed less lipid droplets in the HCD-LD and the high-fat/high-cholesterol diet increased the fat accumulation in the liver. We assumed excess fat accumulation, leading to the alteration of lipid metabolism and liver damage, and dysregulated expression of the lipogenesis marker, SREBP 1, and apoptosis marker, PARP, in the liver. The results showed significant PARP (Figure 5c) and SREBP1 (Figure 5d) expression in HCD vs. CON and reduced expression in HCD-LD and HCD-HD. Elevated HDL could be seen in HCD-LD and HCD-HD. HDL can transfer cholesterol from peripheral tissues to the liver. PON1, the antioxidant enzyme of HDL, determines the proper function of HDL. Both HCD-LD and HCD-HD showed higher PON1 expression than HCD (Figure 5e), which may explain the reduced cholesterol level in the plasma. Since hyperlipidemia is a major risk factor of atherosclerosis, the atherogenic-related proteins were also analyzed. Leukocytes and platelets play important roles in atherosclerosis progression. Adhesion molecules such as ICAM-1 assist monocytes migration. Monocytes bind to ICAM-1 and migrate to the vascular intima, where they differentiate into macrophages. High levels of pro-inflammatory ICAM-1 were expressed in the aorta of HCD, yet ICAM-1 was barely seen in HCD-LD and HCD-HD (Figure 5f). For platelet recruitment and adhesion, expression of VWF was examined. VWF induces a hemostatic platelet plug at the site of vascular injury. The high-fat/high-cholesterol diet enhanced the VWF level in the endothelium of the aorta, while DSW-DOM decreased VWF expression (Figure 5g). Hence, DSW-DOM intake keeps hamsters from HCD-induced endothelial injuries and thrombosis.

### 4.10. DSW-DOM Significantly Delays the Acute Arterial Thrombosis Model of Time to Occlusion

**Figure 6:**
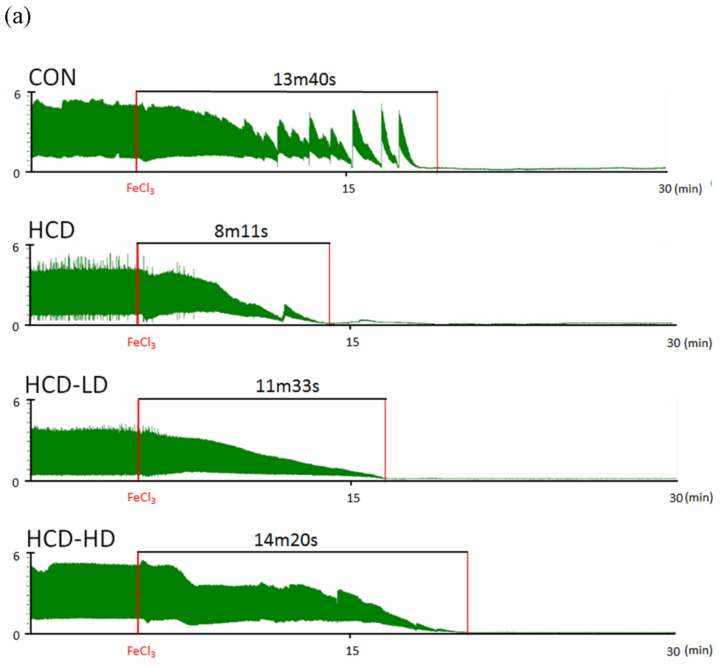

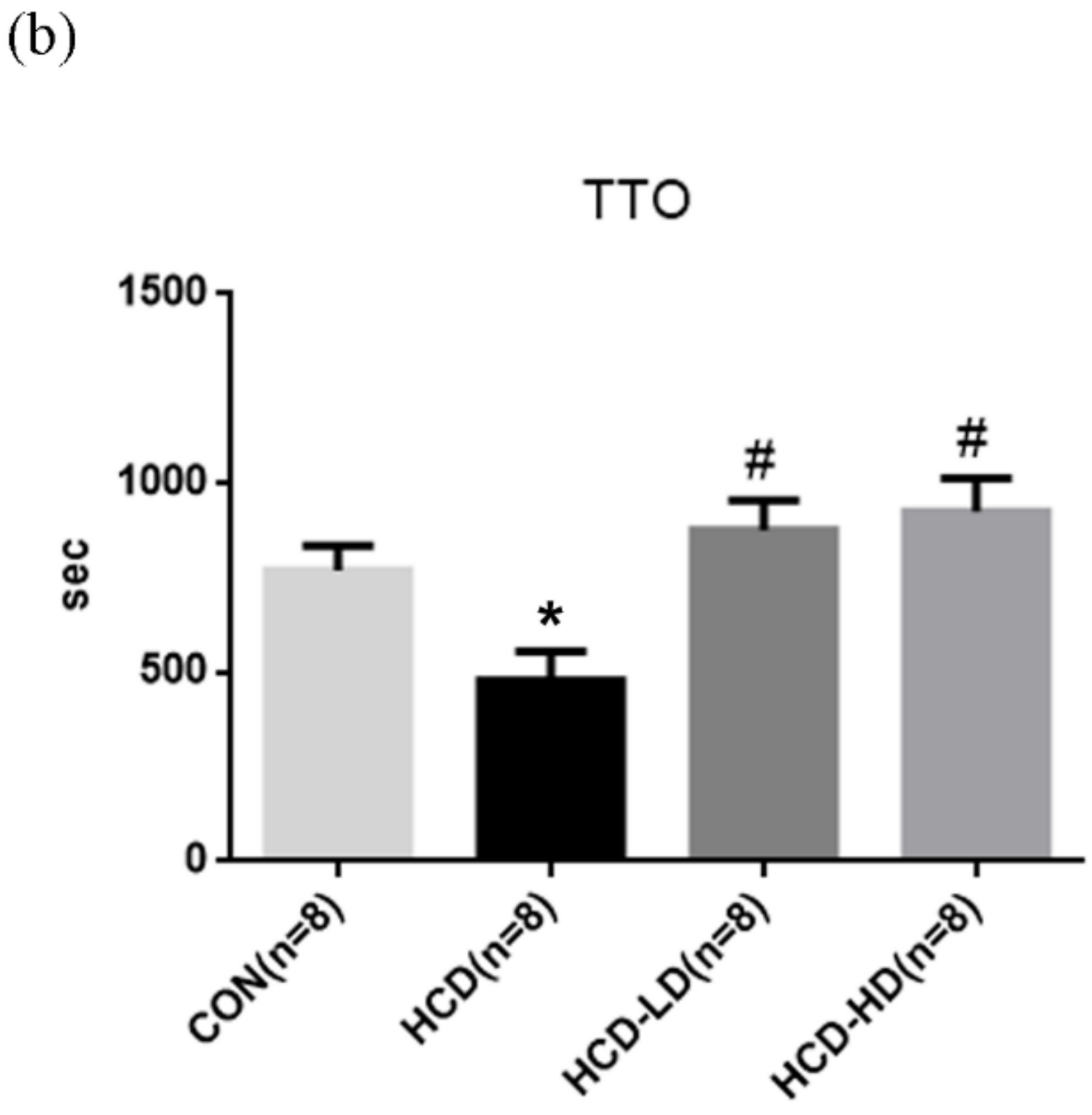
Effect of DSW-DOM on carotid arterial time to occlusion (TTO) in four groups of rats. (**a**) Typical graph of TTO response in each rat of four groups. (**b**) The statistical data in four groups of rats. * *p* < 0.05 vs. CON; # *p* < 0.05 vs. HCD group.

The blood flow over time is shown in Figure 6a, represented by CON, HCD, HCD-LD and HCD-HD. The mean value of TTO of CON was 769.9 ± 65.5 s. In HCD, occlusive thrombus was obtained in 479 ± 76.4 s. In addition, occlusion of HCD-LD and HCD-HD was reached in 875.1 ± 79.6 s and 925 ± 87.9 s after dropping FeCl_3_ (Figure 6b). HCD had the shortest TTO, and both HCD-LD and HCD-HD significantly prolonged the arterial occlusion times.

### 4.11. DSW-DOM Efficiently Inhibits Platelet Adhesiveness Detection in Mesenteric Arterioles

**Figure 7:**
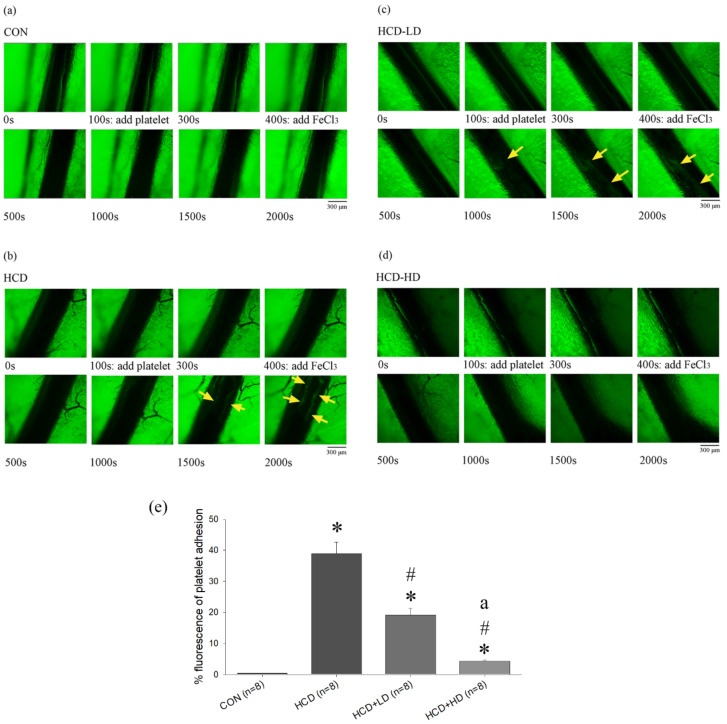
Effect of DSW-DOM on FeCl_3_ induced platelet aggregation in the mesenteric arterioles of CON (**a**), HCD (**b**), HCD-LD (**c**) and HCD-HD (**d**) groups. In the mesenteric arterioles, the green fluorescence of platelets (indicated by yellow arrows) is increased in the HCD and HCD-LD groups. There is little green fluorescence of platelets aggregation in the CON and HCD-HD groups. The statistical data (mean ± SEM, *n* = 8) of the fluorescence intensity is indicated in (**e**). * *p* < 0.05 vs. CON; # *p* < 0.05 vs. HCD group; a *p* < 0.05 vs. HCD + LD group.

FeCl_3_-induced arterial thrombosis and platelet aggregation in mesenteric arterioles were examined by a fluorescent dissecting microscope. FeCl_3_-induced arterial thrombosis over time are shown in Figure 7. In HCD (Figure 7b) and HCD-LD (Figure 7c), multiple platelets could be observed at 1000 s after adding FeCl_3_. A less fluorescence intensity was noted in CON (Figure 7a) and HCD + HD (Figure 7d). At the end of the experiment, HCD presented the highest fluorescence intensity and an obvious thrombosis could be observed (Figure 7e).

### 4.12. DSW-DOM Efficiently Reduces Hepatic CYP2E1, Nfk p65, SREBP-1 and PARP and Increases PON1 Expression

**Figure 8:**
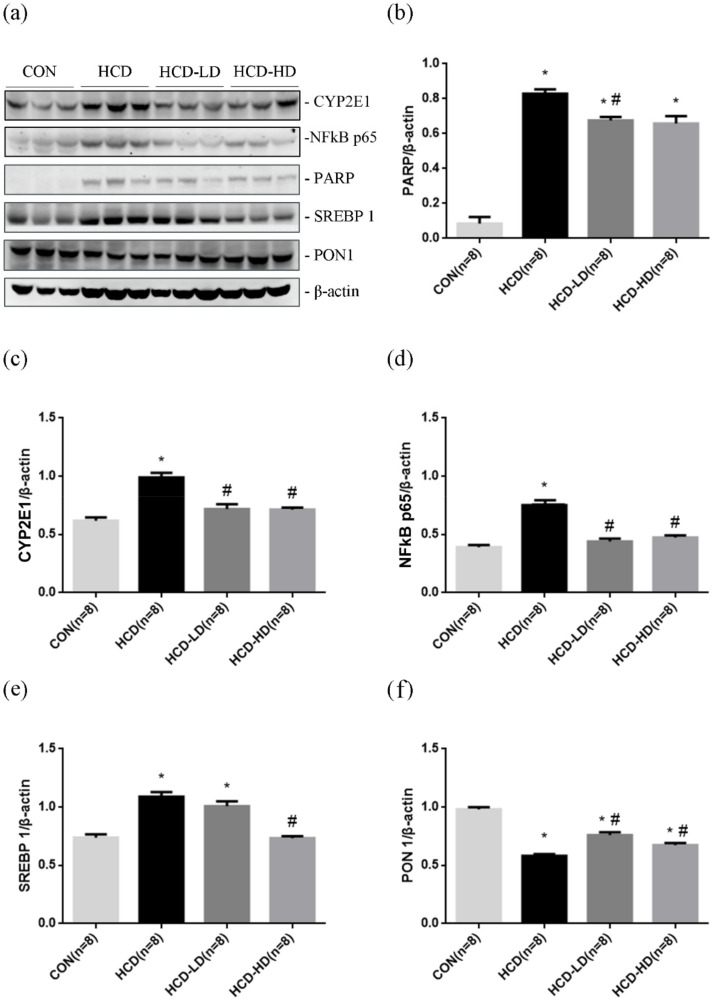
Effect of DSW-DOM on hepatic CYP2E1, NFκB p65, PARP, SREBP 1 and PON1 expression by Western blotting in four groups of rats. (**a**) Typical graphs of the Western blots. The statistical data (mean ± SEM, *n* = 8) are indicated in (**b**) PARP, (**c**) CYP2E1, (**d**) NFκB p65, (**e**) SREBP 1 and (**f**) PON 1 in four groups of hamsters. * *p* < 0.05 vs. CON; # *p* < 0.05 vs. HCD group.

In order to understand how the protective effect of DSW-DOM works on high-fat diet hamsters and quantify the expression of oxidative stress (CYP2E1), inflammation (NFκB p65), lipogenesis (SREBP 1), antioxidant (PON1) and apoptosis-related proteins PARP, Western blotting was carried out. Our data showed that hepatic CYP2E1, NFκB p65, SREBP 1 and PARP were markedly increased in HCD vs. CON (Figure 8a). HCD-LD and LCD-HD significantly downregulated hepatic PARP (Figure 8b), CYP2E1 (Figure 8c), NFκB p65 (Figure 8d) and SREBP 1 (Figure 8e) expression. PON1 with an antioxidant defense mechanism was examined for the purpose of ensuring the proper function of HDL. A high-fat/high-cholesterol diet induced an appreciable decrease in the level of PON1 in HCD. HCD-LD and HCD-HD showed significantly increased PON1 expression (Figure 8f) vs. HCD, which are consistent with the immunohistochemistry results.

## 5. Discussion

Past research has proven the effect of DSW in increasing TEAC and upregulating oxidative stress-related genes in experimental animals with cardiovascular disease [28], hepatic problems [25] and stomach ulcers [43]. Furthermore, DSW with a hardness of 600 and 1200 ppm displayed higher H_2_O_2_ scavenging and hypochlorous acid (HOCl) activity than tap water [43]. Compared with ethanol, either a low or high dose of DSW-DOM, which was obtained from the C18 FF disc, showed better antioxidant capacity. This result suggested administering DSW-DOM is a promising strategy for health promotion as well as disease prevention and treatment.

Excess fat ingestion in one’s daily diet would raise the blood cholesterol and TG levels, which are important risk factors of CVD. In this study, high blood cholesterol and TG levels were induced by the high-fat/high-cholesterol diet (D12108C, 40% kcal fat with 1.25% cholesterol; Research Diet). Fat accumulation increases hepatic free fatty acid (FFA), which either enter mitochondria for β oxidation or become esterified to form TG. These TGs are stored in hepatocytes as lipid droplets or produce VLDL that were later converted into LDL [44]. In the biochemical analysis, the high TC and LDL levels that resulted from the high-fat/high-cholesterol diet were reduced in HCD-HD. Apart from this, the elevated TG content was significantly decreased in both HCD-LD and HCD-HD.

Through histological analyses and TG colorimetric assay, a remarkably reduced hepatic TG content and lipid droplets were found in HCD-LD and HCD-HD. By Western blot analysis, we examined the proteins involved in lipogenesis. Significantly downregulated SREBP 1 in HCD-HD implied decreasing lipid biosynthesis. Accordingly, reduced hepatic lipid droplets resulted from increased fecal TG and decreased TG synthesis. Evidence showed that excess cholesterol can be transferred from peripheral tissues to the liver for catabolism by HDL, a process that is also known as reverse cholesterol transport (RCT) [45]. In the lipid profile, HCD-LD and HCD-HD showed elevated HDL. It has been recognized that HDL has atheroprotective functions against coronary heart disease (CHD). Low HDL levels suggested a high risk of CHD [46]. However, recent studies discovered that increasing HDL did not make expected clinical benefits [47,48]. Besides concentration, other properties of HDL may also be connected with its efficacy [49]. Proper function of HDL depends on the levels of apolipoprotein AI and the antioxidant enzyme PON1 [50]. Dysfunctional HDL was found in plasma of atherosclerotic and diabetic patients with low activity of PON1 [51,52]. In addition, hyperlipidemia decreased PON1 expression in Syrian hamsters [53]. For this reason, we checked the PON1 expression to confirm the quality of HDL. HCD-HD showed increased PON1 expression in Western blot. These results proved that the reduced total cholesterol came from an increasing quality and quantity of HDL by DSW-DOM.

Excess fat intake increases mitochondrial β oxidation of FFA, which relates to the electron transfer chain in mitochondria. Some electrons react with oxygen and thereby produce ROS [54]. Free radicals induce lipid peroxidation and DNA cleavage, which results in cell injury [55]. A high-fat/high-cholesterol diet had induced extraordinarily high blood ROS and plasma MDA in HCD, indicating serious oxidative damage. HCD-LD and HCD-HD showed relatively low ROS and MDA. DSW has been well known as an antioxidant by undefined components. In this experiment, the DSW-DOW supplement successfully diminished the high-fat diet-induced oxidative stress and lipid peroxidation. As a result, DSW-DOM intake perverts oxidative damage.

Several researchers had found that a high-fat diet inhibits the anti-apoptotic signaling pathway and elevates proapoptotic protein expression [56,57]. Oxidative stress as well as elevated proapoptotic proteins contribute to hepatocyte apoptosis [58]. PARP, an indicator of apoptosis, was decreased in HCD-LD and HCD-HD. Hepatic apoptosis and TG accumulation play an important role in the pathogenesis of NASH [59]. Our findings indicated antioxidant capacity and a lipid-lowering effect of DSW-DOM, which prevents or reduces liver disorders.

Increased LDL and oxidative stress raise the risk of LDL oxidation [60]. Ox-LDL stimulates monocytes migration and foam cells development [61]. Foam cells accumulate in the arterial endothelium, form plaque, and ultimately lead to atherosclerosis and thrombosis [62]. Evidence has showed that hyperlipidemia increases the risk of atherosclerosis and thrombosis by regulating platelet activity [63]. Ox-LDL serves as ligands of platelets and activates the platelets [64]. Activated platelets trigger monocyte activation, recruitment and adhesion on the endothelium [65]. To investigate the effect of DSW-DOM on preventing complications of hyperlipidemia, the time to thrombus formation of the tested arteries was examined. In the FeCl_3_-induced arterial thrombosis model, HCD formed an occlusive thrombus in a short period of time, and presented the highest fluorescence intensity in the mesenteric arterioles. In the immunohistochemistry results, HCD presented a high VWF and ICAM-1 in the endothelium of the aorta. VWF is involved in platelet recruitment and adhesion in endothelial cells. On the other hand, ICAM-1 facilitates leukocyte recruitment and vascular complications. Monocytes bind to adhesion molecules and migrate to the intima, where they differentiate into macrophages [49]. Increased expression of these proteins indicated that arteries of HCD were in the early stages of atherosclerosis and vascular inflammation. In contrast, DSW-DOM treatment showed extended TTO, fewer platelet adhesion and less expression of VWF and ICAM-1, which indicated the anti-atherosclerotic effect of DSW-DOM.

DSW has frequently reported to have therapeutic effects on atherosclerosis [20], atopic eczema/dermatitis [21], obesity [22], hyperlipidemia [23,29,30], hypertension [24], liver steatosis [25] and diabetes [26,27] via the action of the rich Mg2+ content. These reports stated that DSW with increased hardness primary from Mg2+ and Ca2+ significantly decreased the atherogenic index [28] and prevented the atherogenesis process [20,30,32]. They suggest that the cardioprotective effects of DSW was ascribed to the minerals in DSW [24,33]. Until now, there is little evidence that indicate the preventive and therapeutic potential of DSW-DOM in biomedical applications. In the present study, our prepared DSW-DOM containing the rich 1295 fraction may exert an antioxidant activity to H_2_O_2_ in our results. Our results also informed that a high-fat diet enhanced cytochrome p450 CYP2E1 and NFκB p65 expression, implicating the increased oxidative stress and inflammation in high-fat diet livers. Our data also evidenced increased blood ROS and plasma MDA elevation in the high-fat diet hamsters. A low and high dose of DSW-DOM efficiently depressed the oxidative stress and inflammation marker, indicating that the 1295 rich fraction in DSW-DOM exerts antioxidant and anti-inflammatory activity to counteract the oxidative stress evoked by hyperlipidemia. Our novel finding of DSW-DOM with the 1295 fraction may provide a new idea to replace the traditional information that DSW-Mg^2+^ plays an important role in the reduction of hyperlipidemia.

## 6. Conclusions

**Figure 9:**
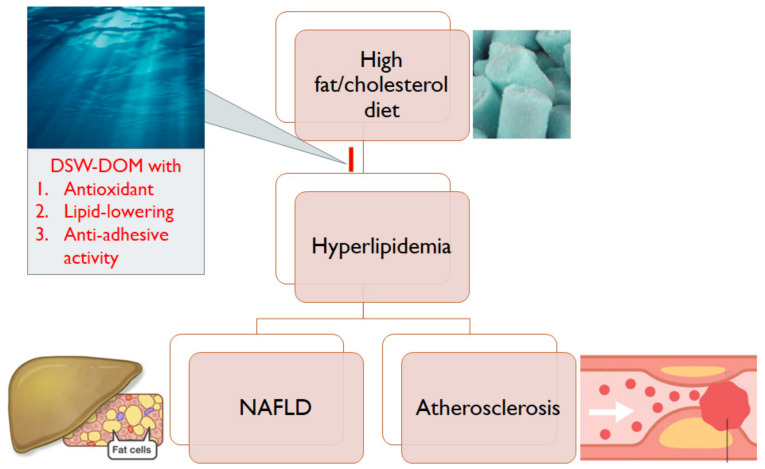
Schematic diagram for demonstrating DSW-DOM’s effect on hyperlipidemia.

Acquired hyperlipidemia comes from an unbalanced diet or insulin resistance. Hyperlipidemia was induced by a high-fat/high-cholesterol diet in the presented study. Excess fat and diet-induced oxidative stress contributed to NAFLD and early stages of atherosclerosis, which may develop into cirrhosis and CVD. The antioxidant capacity, anti-adhesion and hypolipidemic effect of DSW-DOM prevents liver disorders and atherosclerosis (Figure 9).

## Data Availability

The analyzed data sets generated during the study are available from the corresponding author on reasonable request.

## Abbreviations

NAFLD	Non-alcoholic fatty liver disease
*CVD*	Cardiovascular disease
*TG*	Triglyceride
*DSW*	Deep sea water
*DSW-DOM*	Deep sea water-dissolved organic matter
*ox-LDL*	Oxidized low-density lipoprotein
*ROS*	Reactive oxygen species
*MDA*	Malondialdehyde
*LDL-C*	Low-density lipoprotein cholesterol
*VLDL-C*	Very-low-density lipoprotein cholesterol
*TC*	Total cholesterol
*MS*	Metabolic syndrome
*NASH*	Nonalcoholic steatohepatitis
*HCC*	Hepatocellular carcinoma
*LDL*	Low-density lipoprotein
*HDL*	High-density lipoprotein
*AMPK*	AMP-activated protein kinase
*CAD*	Coronary artery disease
*HDL-C*	High-density lipoprotein cholesterol
*TEAC*	Trolox equivalent antioxidant capacity
*LDL-R*	LDL receptor
*IGF-1R*	Insulin-like growth factor 1 receptor
*COX-1*	Cyclooxygenase 1
*HO-1*	Heme oxygenase-1
*CETP*	Cholesteryl ester transport protein
*IR*	Insulin resistance
*CON*	Control group
*HCD*	High-fat/high-cholesterol diet group
*HCD-LD*	High-cholesterol diet with low dose of DSW-DOM
*HCD-HD*	High-cholesterol diet with high dose of DSW-DOM
*C18*	Octadecyl
*ddH_2_O*	Double-distilled water
*CL*	Chemiluminescence
*rcf*	Relative centrifugal field
*FeCl3*	Ferric chloride
*TTO*	Time to occlusion
*rpm*	Revolutions per minute
*PGI2*	Prostacyclin
*NaCl*	Sodium chloride
*Na_2_HPO4*	Sodium hydrogen phosphate
*KCl*	Potassium chloride
*NaHCO3*	Sodium hydrogen carbonate
*HEPES*	4-(2-hydroxyethyl)-1-piperazineethanesulfonic acid
*BSA*	Bovine serum albumin
*PGI_2_*	Prostacyclin
*AM*	Acetoxymethyl
*FP*	Fluorescently-labeled platelets
*H_2_SO4*	Sulfuric acid
*BHT*	Butylated hydroxytoluene
*TBA*	Thiobarbituric acid
*ICAM-1*	Intercellular Adhesion Molecule 1
*PARP*	Poly (ADP-ribose) polymerase
*PON1*	Paraoxonase 1
*SREBP 1*	Sterol regulatory element-binding transcription factor 1
*VWF*	Von Willebrand factor
*HRP*	Horseradish peroxidase
*IgG*	Immunoglobulin G
*PBST*	Phosphate buffer saline tween-20
*DAB*	3′,3′-diaminobenzendine
*RIPA*	Radio immunoprecipitation assay
*BCA*	Bicinchoninic acid
*SDS-PAGE*	Sodium dodecyl sulfate polyacrylamide gel electrophoresis
*PVDF*	Polyvinylidene difluoride
*ECL*	Enhanced chemiluminescence
*HOCl*	Hypochlorous acid
*FFA*	Free fatty acid
*RCT*	Reverse cholesterol transport
*SEM*	Standard error of the mean
*ANOVA*	Analysis of variance
